# *Trichosanthes tricuspidata* Lour. Methanol Extract Exhibits Anti-Inflammatory Activity by Targeting Syk, Src, and IRAK1 Kinase Activity

**DOI:** 10.1155/2019/6879346

**Published:** 2019-12-16

**Authors:** Akash Ahuja, Deok Jeong, Mi-Yeon Kim, Jae Youl Cho

**Affiliations:** ^1^Department of Integrative Biotechnology, Sungkyunkwan University, Suwon 16419, Republic of Korea; ^2^School of Systems Biomedical Science, Soongsil University, Seoul 06978, Republic of Korea

## Abstract

*Trichosanthes tricuspidata* Lour., also known as *T. palmata* Roxb, *T. bracteata* Lam., *T. puber* Blume, and *Modecca bracteata,* is a vine belonging to the Cucurbitaceae family (English name: redball snake gourd). Distributed in China, South and East Asia, and tropical Australia, it has been traditionally used as a medicinal plant for its antifever, laxative, anthelmintic properties and for migraine treatment. In this paper, we examined the effects of *Trichosanthes tricuspidata* Lour. ethanol extract (Tt-ME) *in vitro* and *in vivo*. To confirm the effects of Tt-ME on inflammatory responses, we conducted experimental analyses including level of nitric oxide (NO) production, RT-PCR, and immunoblotting and using a HCl/EtOH-induced gastritis animal model. Tt-ME attenuated the release of NO and decreased mRNA levels of inducible NO synthase (iNOS), TNF-*α*, and IL-6 in lipopolysaccharide- (LPS-) induced macrophages in a concentration-dependent manner. Tt-ME time-dependently suppressed nuclear translocation of nuclear factor kappa B (NF-*κ*B) subunits p50 and p65, activator protein (AP-1) subunits c-Fos and c-Jun, and STAT3 transcriptional activity by inhibiting nuclear translocation of p50, p65, c-Fos, c-Jun, and STAT3. Tt-ME significantly downregulated NF-*κ*B, MAPK, and JAK2 signaling by targeting Syk, Src, and IRAK1 protein kinases. Furthermore, matrix metalloproteinase-9 (MMP-9) expression and cell migration were observed to be downregulated by Tt-ME in LPS-activated macrophages. *In vivo* studies on Tt-ME also produced similar trends in Hcl/EtOH-induced gastritis mouse models by inhibiting proinflammatory cytokines and the inflammatory signaling pathway. Our results strongly suggest that Tt-ME exerted anti-inflammatory activity in LPS-stimulated macrophages and mouse models of acute inflammatory disease.

## 1. Introduction

Inflammation is an adaptive response which can be triggered by microbial injection (bacterial infection) or tissue injury and activates toll-like receptors (TLRs) and nucleotide-binding oligomerization-domain proteins (NLRs). This innate immune response is mediated by macrophages and mast cells, resulting in activation of inflammatory signaling pathways, chemokines, cytokines, etc. [1]. Most previous studies have been focused on lipopolysaccharides (LPS), which are known to activate an inflammatory response in macrophages, dendritic cells, and neutrophils, resulting in cancer invasion and angiogenesis [2–4]. The role of TLR4, the receptor for LPS, has been studied in various inflammation-related disorders [5, 6]. Activation of inflammation triggers intracellular signaling, resulting in activation or overexpression of proinflammatory transcription factors such as NF-*κ*B, STAT3, and AP-1 and enhancing the secretion of inflammatory molecules such as NO and PEG_2_ and the production of cytokines and chemokines [7, 8]. Activated NF-*κ*B results in activation of proto-oncoprotein Src, which can directly phosphorylate STAT3 [9]. STAT3 and NF-*κ*B can stimulate highly proliferative and proangiogenic genes [10, 11]. Cytokines and growth factors such as IL-6 and fibroblast growth factor (FGF) are known to activate STAT3, which promotes oncogenesis and often activates inflammation or cancer [12, 13].

The genus *Trichosanthes tricuspidata* Lour., also known as *T. palmata* Roxb, *T. bracteata* Lam., *T. puber* Blume, or *Modecca bracteata*, is an attractive vine belonging to the family Cucurbitaceae (English name: redball snake gourd) and believed to be native to China, South and East Asia, and tropical Australia. According to the literature, the plant has been traditionally used for its antifever, laxative, anthelmintic properties and for migraine treatment [14]. Previous studies have been focused on isolating the chemical constituents of *T. tricuspidata*, which include trichotetrol isolated from the roots and 14 cucurbitane compounds and glycosides isolated from the fruit. Two new cucurbitacins have also been reported: tricuspidatin and 2-O-glucocucurbitacin [15]. The plant has traditionally been considered to be a medicinally important plant with multiple pharmacological activities. However, pharmacological studies on *T. tricuspidata* are still limited, and the detailed mode of action and mechanism of its anti-inflammatory activity remain largely unknown.

Therefore, in order to study the anti-inflammatory mechanism of *T. tricuspidata*, we examined its inhibitory effects on the mRNA expression profiles of proinflammatory cytokines as well as the underlying inflammatory signaling pathways in RAW264.7 macrophages. Particularly, we found the effect of Tt-ME on anti-inflammatory processes, cell migration, and invasion. Furthermore, we performed *in vivo* experiments on HCl/EtOH-induced gastritis in mouse models, which produced a similar trend in the inhibition of proinflammatory cytokines and the inflammatory signaling pathway.

## 2. Materials and Methods

### 2.1. Materials

Methanol extract (80%) from the leaves of *Trichosanthes tricuspidata* Lour. (Tt-ME) was obtained from the Korea Plant Extract Bank (Cheongju, South Korea). Dimethyl sulfoxide (DMSO), L-N^G^–nitroarginine methyl ester (L-NAME), polyethylene imidazole (PEI), lipopolysaccharide (LPS, *Escherichia coli* 0111:B4), (3-4,5-dimethylthiazol-2-yl)-2,5-diphenyltetrazolium bromide (MTT), sodium dodecyl sulfate (SDS), and ranitidine were purchased from Sigma Chemical Co. (St Louis, MO, USA). Fetal bovine serum (FBS), phosphate-buffered saline (PBS), TRIzol reagent, Dulbecco's modified Eagle's medium (DMEM), and Roswell Park Memorial Institute (RPMI) 1640 were purchased from GIBCO (Grand Island, NY, USA). RAW264.7 cells from mice (BLAB/c, ATCC number TIB-71) and human embryonic kidney 293 cells (HEK293) (ATCC number CRL-1573) were purchased from ATCC (Rockville, MD, USA). Phosphorylated and total protein antibodies against p65, p50, I*κ*B*α*, IKK*α*/*β*, Syc, Syk, STAT3, JAK, and *β*-actin were purchased from Cell Signaling (Beverly, MA, USA). RT-PCR primers for iNOS, TNF-*α*, COX-2, and GAPDH were synthesized from Bioneer Inc. (Daejeon, Republic of Korea).

### 2.2. Mice

ICR mice (male, 6–8 weeks old) were purchased from Dachan Biolonk (Osong, Korea). Mice were maintained in animal care facilities at Sungkyunkwan University (SKKU) following the Institute/University Animal Care and Use guidelines.

### 2.3. Cell Culture and Drug Treatment Conditions

RAW264.7 (mouse-derived) cell lines were cultured and maintained at 37°C with 5% CO_2_ in RPMI 1640 containing 10% heat-inactivated FBS, L-glutamine, and 100 U/ml antibiotics (penicillin and streptomycin). HEK293 cells were cultured at 37°C with 5% CO_2_ in DMEM supplemented with 5% heat-inactivated FBS, L-glutamine, and 100 U/ml antibiotics (penicillin and streptomycin). For cell-based experiments, Tt-ME stock solution (200 *μ*g/ml) was prepared in dimethyl sulfoxide (DMSO). For animal experiments, Tt-ME (200 mg/ml) was dissolved in 0.5% sodium carboxymethyl cellulose (sodium CMC), and drug treatment was performed as previously described [16, 17].

### 2.4. Isolation of Peritoneal Macrophage

Peritoneal macrophages were isolated from male mice (C57BL/6). Thioglycollate broth (4%) (Difco Laboratories, Detroit, MI) was injected in the peritoneum for four days as described by others [18]. Peritoneal cells were isolated and washed three times and suspended in RPMI containing 10% FBS with antibiotics prior to being counted and plated in culture for experiments.

### 2.5. Expression Vector Construction

Expression vectors were constructed by using restriction enzyme treatment of genes and vectors. PCR products of the genes were amplified and cloned into the blunt ends of the cloning vector, using standard protocols with competent *E. coli* (DH5*α*). Flag-MyD88, CFP-TRIF, Myc-Syk, and HA-Src were used as reported. Luciferase constructs that contained NF-*κ*B binding sites were used as previously reported [18, 19]. All constructs were confirmed by DNA sequencing.

### 2.6. Nitric Oxide Production

RAW264.7 cells (1 × 10^6^ cells/ml) and peritoneal macrophages (5 × 10^6^ cells/ml) were plated and preincubated in a CO_2_ incubator. The cells were then pretreated with Tt-ME (0–200 mg/ml) or L-NAME for 30 min and then treated with LPS (1 *μ*g/ml) for 24 h. The amount of nitric oxide produced was determined using the Griess reagent as previously described [20].

### 2.7. Cell Viability

RAW264.7 cells (1 × 10^6^ cells/ml) or peritoneal macrophages (5 × 10^6^ cells/ml) were preincubated and then treated with Tt-ME (0–200 mg/ml) for 24 h. The cells were then treated with 10 *μ*l of MTT solution (10 mg/ml in PBS, pH 7.4) for 4 h [19, 20]. The reaction was stopped by adding 15% sodium dodecyl sulfate (100 *μ*l). The samples were then incubated for an additional 24 h. The absorbance was detected at 570 nm and calculated with respect to the nontreated control groups.

### 2.8. mRNA Isolation and Semiquantitative RT-PCR

RAW264.7 cells were pretreated with Tt-ME (50, 100, 150, and 200 *μ*l/ml) for 30 min and then supplemented with LPS (1 *μ*g/ml) for 6 h at 37°C with 5% CO_2_. Total RNA was isolated by using TRIzol reagent (Gibco BRL) according to manufacturer instructions. RNA samples were then quantified, and one microgram of total RNA was reverse-transcribed with M-MuLv Reverse Transcriptase (New England Biolabs) according to manufacturer instructions. Semiquantitative RT-PCR was performed as reported, and the gel images were documented by a gel doc and images were quantified using ImageJ [21]. Relative expression levels of inflammatory genes determined by semiquantitative and quantitative real-time PCR were calculated with respect to GAPDH as an internal control. Primer sequences for RT and real-time PCR are listed in Table 1.

### 2.9. Luciferase Reporter Gene Activity Assay

HEK293 cells were transfected with plasmids expressing NF-*κ*B-luciferase (1 *μ*g/ml) or AP-1 (Activator Protein-a), *β*-galactosidase (0.1 *μ*g/ml), and Flag-MyD88 (1 *μ*g/ml) for 24 h using PEI in a 24-well plate. Cells were subsequently treated with Tt-ME (0–200 *μ*g/ml) for 24 h. Cells underwent three rounds of freezing and thawing. Cell lysates were used to measure NF-*κ*B and AP-1-mediated luciferase activity with a luciferase assay system as previously reported [22].

### 2.10. Preparation of Whole Cell Lysates and Nuclear Extracts

RAW264.7 cells were harvested with PBS and lysed with ice cold lysis buffer (20 mM Tris-HCL, pH 7.4, 2 *μ*M EDTA, 2 *μ*M EGTA, 50 *μ*M glycerol phosphate, 1 mM DTT, 2 *μ*g/ml aprotinin, 2 *μ*g/ml leupeptin, 1 *μ*g/ml pepstatin, 50 mM PMSF, 1 mM benzamide, 2% Triton X-100, 10% glycerol, 0.1 mM sodium vanadate, 1.6 mM pervanadate, and 10 mM NaF). The isolated cell lysate was then centrifuged at 12000 rpm for 5 mins at 4°C and stored at −70°C for further use. Nuclear cell lysate was also prepared as previously described [23]. The cells were lysed in ice cold lysis buffer A (400 *μ*l) containing 10 mM HEPES pH 7.8, 10 mM KCL, 2 mM MgCl_2_, 0.1 mM EDTA, 1 mM DTT, 0.1 mM PMSF, 2 *μ*g/ml leupeptin, and 2 *μ*g/ml aprotinin and centrifuged at 12,000 rpm for 1 min. The supernatant was then discarded, and the cell lysate was again washed with cell lysis buffer containing 252 *μ*l of 10% NP-40. The cell lysate was vigorously vortexed and centrifuged at 14000 rpm for 1 min at 4°C, with the resulting supernatant being stored at −70°C as cytosol samples. The remaining cell pellets were again washed with lysis buffer at 14000 rpm for 1 min, and the supernatant was discarded. The cell pellets were suspended with nuclear extraction buffer (50 *μ*l) containing 10 mM HEPES pH7.8, 50 mM KCl, 400 mM NaCl, 0.1 mM EDTA, 1 mM DTT, 0.1 mM PMSF, 2 *μ*g/ml leupeptin, 2 *μ*g/ml aprotinin, and 10% glycerol. The solution was incubated in ice for 20 min and vortexed vigorously at five-minute intervals. The cells were then centrifuged at 14000 rpm at 4°C for 5 min. The supernatant was transferred to fresh tubes and stored at −70°C until use.

### 2.11. Western Blotting of Phospho and Total Proteins

Whole cell extracts were prepared, and protein concentration was determined by Bradford reagent (Bio-Rad). Whole cell extracts (20 *μ*g/ml) were then fractionated via 10% sodium dodecyl sulfate-polyacrylamide gel electrophoresis (SDS-PAGE). Gels were run at 100 V for 2 h and then transferred to a polyvinylidene difluoride (PVDF) membrane (Millipore Corp, Billerica, MA, USA). After the transfer, the nitrocellulose blots were incubated for 1 h with primary antibodies diluted in TBS/Tween 20 (0.0075%). Mouse monoclonal antibodies directed against p50, p65, I*κ*B*α*, IKK*α*/*β*, AKT, p85, ERK, JNK, JAK2, Syk, Src, MEK1/2, MKK3/4, TAK1, IRAK1, MMP-2, and *β*-actin (Cell Signaling) were used to detect phosphorylated and total protein. Following incubation with primary antibodies, blots were washed three times with TBS/Tween 20 before a 1 h incubation with secondary anti-mouse or anti-rabbit antibodies. After secondary antibody treatment, blots were again washed with TBS/Tween 20 and then processed for detection using a chemiluminescence system. Proteins were visualized using an enhanced chemiluminescent (ECL) system (Amersham, LiTte Chalfont, Buckinghamshire, UK) as previously reported [24].

### 2.12. EtOH/HCl-Induced Gastritis in Mice Models

Mice were divided into four groups of five: control, EtOH/HCl, Tt-ME 200 mk/kg, and ranitidine 40 mg/kg. Tt-ME (200 mg/kg) and ranitidine (40 mg/kg), dissolved in 0.5% CMC, were administered orally after 24 h of fasting, followed by 60% EtOH/150 mM HCl, also administered orally for 1 h. The mice were then dissected, the stomachs removed and washed with PBS. After washing, the stomachs were opened, and the gastric lesions were documented by spreading them. The stomach samples were then stored in −70°C for protein and gene expression analysis.

### 2.13. Cell Migration Assay

Briefly, confluent RAW264.7 cells were treated with Tt-ME (100 and 200 *μ*g/ml) for 30 minutes and then stimulated with LPS (1 *μ*g/ml). The plates were scratched by manual scratching with a 200 ml pipette tip. Subsequently, the cells were washed with PBS and incubated at 37°C in complete media. At the indicated time points, phase contrast images at specific wound sites were taken, and the images were then quantified using ImageJ software.

## 3. Results

### 3.1. Effect of Tt-ME on LPS-Induced RAW264.7 and Peritoneal Macrophages

**Figure 1:**
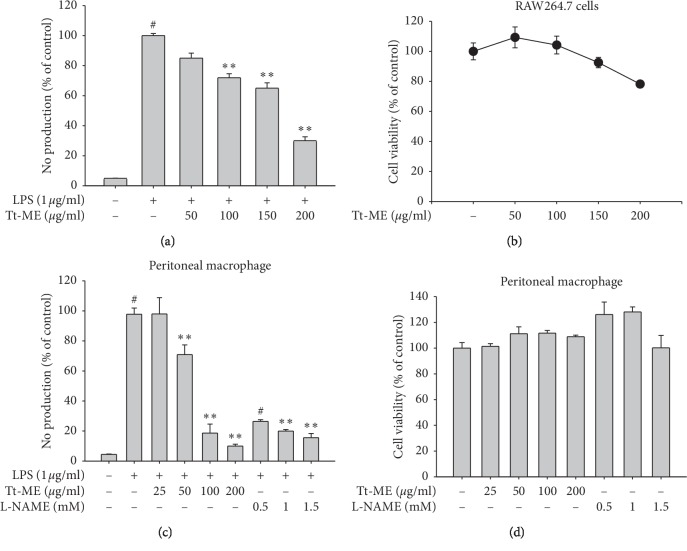
Effect of Tt-ME on LPS-induced RAW264.7 and peritoneal macrophages. (a) Macrophages like RAW264.7 were pretreated with increasing concentrations of Tt-ME for 30 minutes before treatment with LPS for 24 h. The NO level in the solution was then determined. (b) RAW264.7 cells were treated with indicated concentrations of Tt-ME for 24 h after which viability of the Tt-ME-treated cells was then determined using an MTT assay. (c) Peritoneal macrophages were obtained from peritoneal exudates of thioglycollate-injected mice. NO levels in LPS-treated peritoneal macrophages in the presence or absence of Tt-ME and L-NAME were determined using the Griess assay. (d) Cell viability of peritoneal macrophages was determined in Tt-ME- and L-NAME-treated samples using an MTT assay. The results are representative of three independent experiments and are expressed as mean ± SD. ^*∗*^*p* > 0.05 versus controls.

We examined whether *Trichosanthes tricuspidata* Lour. methanol extract (Tt-ME) inhibits inflammatory mediators such as the supernatant NO on LPS-induced RAW264.7 cells. As shown in Figure 1(a), NO levels were significantly increased in cells treated with LPS (1 *μ*g/ml), compared with nontreated control groups. However, pretreatment of cells with different concentrations of Tt-ME (0–200 *μ*g/ml) decreased the NO production, a decrease that was statistically significant at a Tt-ME concentration of 200 *μ*g/ml with an 80% decrease in NO production stimulated by LPS. Furthermore, we confirmed whether Tt-ME produces cytotoxic effects using the MTT cell viability assay. The result of the toxicity study of Tt-ME indicated that the extract treated with varying concentrations of Tt-ME (0–200 *μ*g/ml) did not yield any sign of toxicity in any of the groups during or after treatment (Figure 1(b)). These results indicate that Tt-ME shows an anti-inflammatory effect without cytotoxicity at 200 *μ*g/ml. Therefore, a Tt-ME concentration of 200 *μ*g/ml was selected as the optimum dose in this study.

To further confirm the anti-inflammatory effects of Tt-ME, we used peritoneal macrophages and studied NO and MTT during incubation for 24 h. The cells treated with LPS alone markedly increased the NO production up to 100% (Figure 1(c)). However, the Tt-ME (at concentrations of 25, 50, 100, and 200 *μ*g/ml) dose-dependently decreased the NO production, culminating in a 90% decrease in the 200 *μ*g/ml Tt-ME-treated group. Moreover, cell viability was also observed to be <100% in Tt-ME-treated groups (at all four concentrations) (Figure 1(d)). In the parallel experiment, L-NAME, an NO synthase inhibitor, significantly suppressed the NO production with no cytotoxicity levels in peritoneal macrophages (Figures 1(c) and 1(d)).

### 3.2. Anti-Inflammatory Effects of Tt-ME on Gene Expression of Cytokines and Nuclear Translocation of NF-*κ*B, AP-1, and STAT3 Transcription Factors

**Figure 2:**
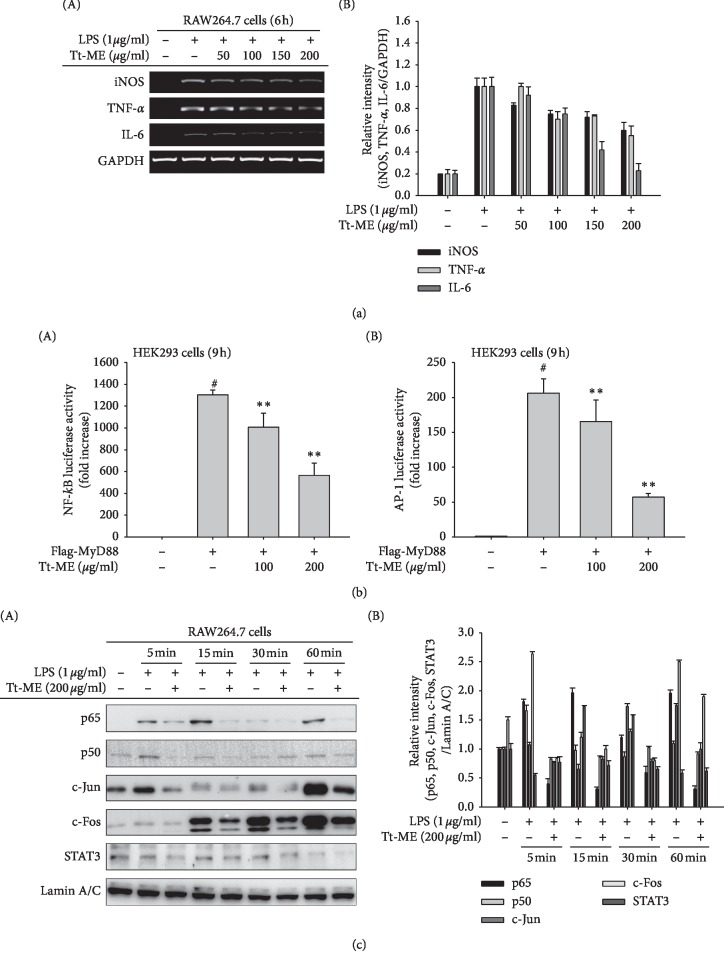
Anti-inflammatory effects of Tt-ME on gene expression of cytokines and nuclear translocation of NF-*κ*B, AP-1, and STAT3 transcription factors. (a) RAW264.7 cells were treated with increasing concentration of Tt-ME for 30 min and then treated with LPS (1 *μ*g/ml) for 6 h followed by isolation of RNA and generation of cDNA. Quantitative PCR levels of iNOS, TNF-*α*, and IL-6 gene expression were determined with GAPDH as a housekeeping gene. (b) Luciferase reporter assay. HEK293T cells were transfected over night with NF-*κ*B, AP-1 luciferase vector, and *β*-gal expression vector constructs and then treated with the indicated dose of Tt-ME overnight. The levels of NF-*κ*B and AP-1-mediated luciferase were determined by using a luminometer. (c) RAW264.7 cells were pretreated with Tt-ME (200 *μ*g/ml) for 30 minutes and then treated with LPS (1 *μ*g/ml) for the indicated period of time. Representative western blot analyses of NF-*κ*B subunits (p50 and p65), AP-1 subunits (c-Fos and c-Jun), and STAT3 were performed, and the nuclear translocation levels were calculated by densitometric analysis of band intensities. Data represent mean ± SD, replicates of three (^*∗*^*p* < 0.05; ^*∗∗*^*p* < 0.01 students *t*-test). The experiment was repeated, and similar results were observed.

In the present study, RAW264.7 macrophage-like cells were dose-dependently pretreated with Tt-ME (ranging from 50 to 200 *μ*g/ml) and activated with LPS (1 *μ*g/ml) for 6 h. Gene expression profiles of iNOS, TNF-*α*, and IL-6 were then detected by RT-PCR. As shown in Figure 2(a), the expression profiles of iNOS, TNF-*α*, and IL-6 showed a distinct increase in expression levels in LPS-treated groups, whereas pretreatment with Tt-ME (200 *μ*g/ml) inhibited the LPS-induced gene expression of iNOS, TNF-*α*, and IL-6. Taken together, our results indicate that Tt-ME inhibits the LPS-directed release of proinflammatory cytokines by downregulating gene transcription. NF-*κ*B and AP-1 are key transcription factors that control the activity of inflammatory mediators induced by LPS [25]. To examine the anti-inflammatory role of Tt-ME, we then tested the transcriptional activity of NF-*κ*B and AP-1 by luciferase reporter gene assay using HEK293T cells. As shown in Figure 2(b), Tt-ME dose-dependently decreased the NF-*κ*B and AP-1 luciferase activity, suggesting that Tt-ME could regulate multi-inflammatory signaling pathways. To further confirm, we checked the inhibitory effects of Tt-ME on the nuclear translocation of NF-*κ*B subunits p50 and p65, AP-1 subunits c-Fos and c-Jun, and STAT3 time-dependently in RAW264.7 cells 60 min after LPS stimulation. Nuclear levels of p65, p50, c-Fos, c-Jun, and STAT3 were markedly increased in LPS-treated groups, whereas Tt-ME significantly suppressed the nuclear translocation of p65, p50, c-Fos, c-Jun, and STAT3 (Figure 2(c)). These data suggest that Tt-ME exhibits anti-inflammatory qualities through inhibition of the nuclear translocation of NF-*κ*B, AP-1, and STAT3 transcription factors.

### 3.3. Effect of Tt-ME on the Inflammatory Signaling Pathways

**Figure 3:**
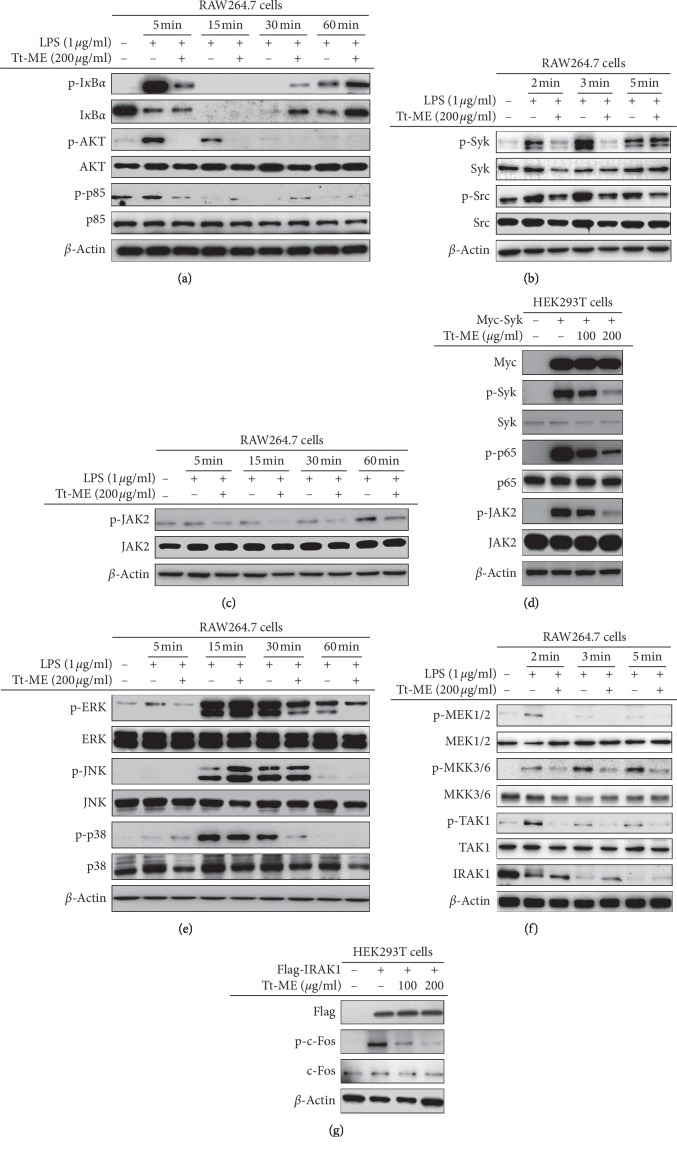
Effect of Tt-ME on the inflammatory signaling pathways. (a, c, e) RAW264.7 cells were treated with 200 *μ*g/ml Tt-ME for 30 minutes and supplemented with untreated or treated LPS (1 *μ*g/ml) for indicated periods. Phosphorylation of I*κ*B*α*, p85, ERK, JNK, p38, and JAK2 was assessed by immunoblotting. (b, f) RAW264.7 cells were treated with 200 mg/ml Tt-ME for 30 minutes and supplemented with LPS for a short duration (2, 3, and 5 minutes), after which phospho and total forms of upstream signaling molecules of NF-*κ*B and MAPK were assessed by western blotting. The results are representative of three independent experiments. (d, g) Myc-Syk and Flag-IRAK1 plasmid construct were transfected into HEK293T cells for 18 h. The cells were then treated in the presence or absence of Tt-ME for 18 h. Whole cell lysates were then prepared, and immunoblotting was performed. The results are representative of three independent experiments.

Several reports have indicated that TLR4 plays a key role in recognition of LPS, which results in activation of intracellular inflammatory cascades by recruiting MyD88 and activation of NF-*κ*B and AP-1 complex, further resulting in induction of proinflammatory genes such as TNF-*α*, COX-2, iNOS, IL-6, and IL-1*β* [22, 26]. Similarly, STAT3 has been known to share a putative conserved region within NF-*κ*B promoters [27]. Therefore, we hypothesized that LPS might activate the induction of the NF-*κ*B, AP-1, and STAT3 signaling cascade. The inhibitory patterns of Tt-ME were investigated in RAW264.7 macrophages stimulated with LPS (1 *μ*g/ml) time-dependently (ranging from 5 to 60 min). As shown in Figure 3(a), Tt-ME (200 *μ*g/ml) pretreatment inhibited the phosphorylation of I*κ*B*α* at 5 min followed by parallel degradation of I*κ*B*α* at 30 min. Studies suggest that NF-*κ*B regulates the transcriptional activity of AKT by parallel phosphorylation and degradation of I*κ*B*α* [28]. Phosphorylation of AKT on S473 residue at 5 to 15 min and regulatory subunit p85 was strongly suppressed by Tt-ME at 5 min in comparison with LPS-stimulated RAW264.7 macrophages. We further explored the upstream signaling pathway of p85 to find the target of Tt-ME. As shown in Figure 3(b), the phosphorylation level of NF-*κ*B upstream kinases such as Syk and Src was strongly downregulated by Tt-ME (200 *μ*g/ml) at 2 and 3 min. Furthermore, previous data showed that Tt-ME inhibits the STAT3 signaling pathway. To explore the target molecule on the STAT3 pathway, we confirmed phospho JAK2 levels on LPS-induced inflammatory responses. As a result, the phospho JAK2 level was inhibited by 200 *μ*g/ml of Tt-ME (Figure 3(c)). Finally, we evaluated the Syk overexpression using HEK293T cells to confirm the effect of Tt-ME. As shown in Figure 3(c), the phosphorylation levels of Syk and p65 were increased by Syk overexpression. On the other hand, phosphorylation levels were inhibited by Tt-ME (200 *μ*g/ml) in a dose-dependent manner. Interestingly, overexpression of Syk induced the phospho JAK2 level, while the phosphorylation level of JAK2 was reduced by Tt-ME. These data indicated that Tt-ME exhibits an anti-inflammatory effect by targeting Syk and Src on NF-*κ*B and the STAT3 signaling pathway.

We further explored the response of Tt-ME on AP-1 signaling pathways, as these transcription factors are known to be activated by the same multitude of stimuli [29, 30]. Pretreatment with Tt-ME (200 *μ*g/ml) for 30 min followed by LPS treatment (5 to 60 min) reduced the phosphorylation of ERK at 5 min (Figure 3(e)). However, there was no significant inhibition observed in the phosphorylated and total forms of JNK and p38, suggesting that Tt-ME could regulate upstream of ERK. We next focused on upstream signaling to find the target molecule through which Tt-ME governs its anti-inflammatory effects. To study this, we examined whether Tt-ME modulates LPS-stimulated ERK upstream kinases MEK1/2, MKK3/6, TAK1, and IRAK1. As shown, stimulation of Tt-ME for 30 min and LPS stimulation for 2, 3, and 5 min inhibited the phosphorylation of MEK1/2, MKK3/6, and TAK1. Interestingly, Tt-ME inhibited the degradation of IRAK1 which is an upstream protein on AP-1 signaling pathways at 2 min (Figure 3(f)). To confirm that this protein is the Tt-ME target, levels of IRAK1 were examined by overexpression in HEK293T cells as previously reported [31]. Western blotting analysis showed that overexpression of IRAK1 highly phosphorylated c-Fos in the case of AP-1 signaling, whereas Tt-ME dose-dependently (100 and 200 *μ*g/ml) inhibited c-Fos phosphorylation (Figure 3(g)). These data indicate that Tt-ME exhibits anti-inflammatory effects by targeting IRAK1.

### 3.4. Tt-ME Prevents LPS-Induced Migration Abilities in RAW264.7 Macrophages

**Figure 4:**
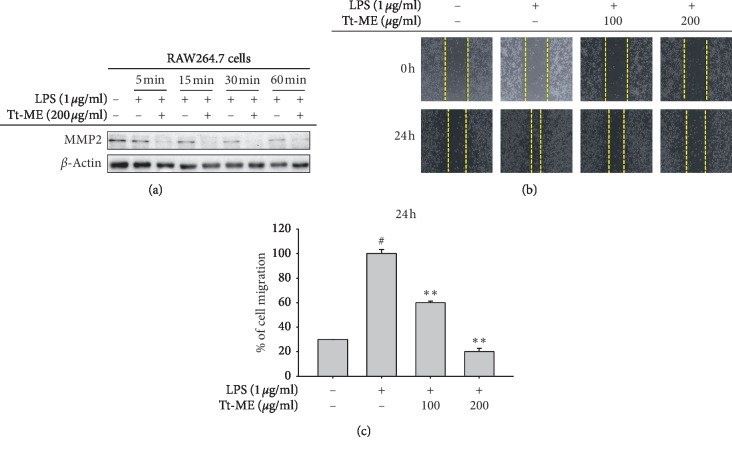
Tt-ME prevents LPS-induced migration abilities in RAW264.7 macrophages. (a) RAW264.7 macrophages were pretreated with Tt-ME (200 *μ*g/ml) for 30 minutes and then treated with LPS (1 *μ*g/ml) for the indicated period. Western blot analysis was performed to confirm MMP-2 levels. (b) RAW264.7 cells were scratched and then treated with Tt-ME for 30 minutes and then treated with LPS (1 *μ*g/ml) for 24 h. Representative images are shown at indicated time points. (c) Results were quantified using ImageJ software, and error bars represents SD of the means of three independent experiments performed in triplicates. Data are shown as the mean ± SD of three independent experiments. ^*∗*^*p* < 0.01; ^*∗∗*^*p* < 0.05.

To further confirm the role of Tt-ME in LPS-induced enhancement of migration, we next determined whether pretreatment of Tt-ME (200 *μ*g/ml) for 30 min, followed by LPS activation, inhibited the migration behavior of RAW264.7 cells. Western blotting indicated that 200 *μ*g/ml Tt-ME time-dependently inhibited MMP-2 levels (Figure 4(a)). The inhibitory effect of Tt-ME was subsequently studied with the help of a cell migration assay (Figures 4(b) and 4(c)). The LPS-treated group showed a very high degree of cell migration time-dependently, but migration was significantly decreased in Tt-ME-treated groups. These results confirm that Tt-ME exhibits antimigratory behavior.

### 3.5. Inhibitory Effects of Tt-ME on HCL/EtOH-Induced Gastritis

**Figure 5:**
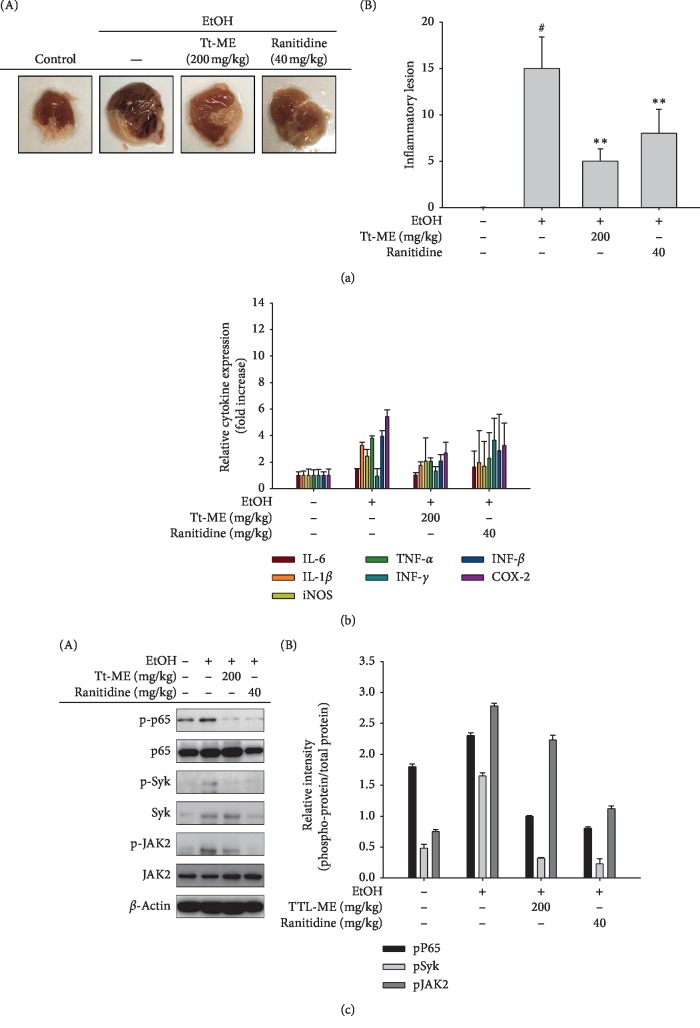
Inhibitory effects of Tt-ME on HCL/EtOH-induced gastritis. (a) Mice were orally administered Tt-ME (0 and 200 mg/kg) or ranitidine (40 mg/kg) every day for 3 days before oral administration of HCl/EtOH. One hour after oral administration of HCl/EtOH, stomachs of the mice were excised, and the gastritis lesions in the stomachs were photographed using ImageJ software. (b) mRNA expression levels of IL-6, IL-1*β*, iNOS, TNF-*α*, IFN-*γ*, IFN-*α*, and COX-2 from the stomach tissues of mice treated with HCl/EtOH were determined by quantitative real-time PCR. (c) Western blot analyses for phosphorylated and total protein levels of p65, Syk, and JAK2 were performed in stomach lysates, and the relative protein levels were analyzed by ImageJ software (right panel). Data are shown as mean ± SEM, *n* = 3. ^*∗*^*p* < 0.01; ^*∗∗*^*p* < 0.05.

In order to substantiate the finding, we used a gastritis (HCL/EtOH)-induced mouse model to analyze the effect of Tt-ME (200 mg/kg) in comparison with ranitidine (40 mg/kg), a universal drug used to treat gastric ulcers. Tt-ME treatment (200 mg/kg) significantly reduced the gastric inflammatory lesions (Figure 5(a)). Similarly, Tt-ME (200 mg/kg) also reduced the major cytokine gene expression profiles of IL-6, IL-1*β*, iNOS, TNF-*α*, INF-*γ*, INF-*β*, and COX-2 (Figure 5(b)). Immunoblotting analysis also inhibited the phosphorylation of p65, Syk, and JAK2. These *in vivo* results were in agreement with *in vitro* results (Figure 5(c)). Our overall results demonstrate that administration of Tt-ME significantly reduced gastric lesions by inhibiting inflammatory signaling pathways.

## 4. Discussion

Plant-derived natural compounds have a long history of treating infectious diseases and contribute to the health of millions of people worldwide [32]. These natural components can be derived from leaves, flowers, roots, fruits, etc. In this paper, we have studied the anti-inflammatory effect of *T. tricuspidata* Lour. (Tt-ME), a methanol compound isolated from the root of *T. tricuspidata*. In folk herbal medicine, the plant was used for the treatment of epilepsy, lung disease, cough, atopic dermatitis, and smallpox [33, 34]. Previous studies that documented the phytochemical constituents of the methanolic extract of *T. tricuspidata* observed the presence of carbohydrates, alkaloids, phenolic compounds, saponin, fats, and oils [33]. The phytochemical constituents and ethno-pharmacological evidence suggest that *T. tricuspidata* methanolic extract plays a crucial role in anti-inflammatory behavior in macrophages. In the current work, we demonstrated the anti-inflammatory mechanism of Tt-ME on LPS-induced RAW264.7 macrophages.

LPS has been known to induce the overproduction of nitric oxide (NO) by inducing the iNOS gene [35]. Moreover, an increase in NO production leads to activation of macrophages and causes inflammation [36]. Therefore, we first identified the effect of Tt-ME on LPS-induced NO production in RAW264.7 macrophages, which was that Tt-ME (25, 50, 100, and 200 *μ*g/ml) effectively inhibited the NO production in a dose-dependent manner (Figure 1(a)). In addition, an MTT assay confirmed that the NO production inhibition effect was not due to the induction of cytotoxicity of Tt-ME (Figure 1(b)). The NO inhibition and cytotoxic effect was also confirmed in peritoneal macrophages isolated from mice. Tt-ME showed similar patterns of dose-dependent inhibition of NO production levels, whereas positive controls L-NAME also inhibited NO production (Figures 1(c) and 1(d)).

Macrophages, when activated by external stimuli (LPS) through binding the CD14/TLR4/MD receptor complex, participate in the acute phase response and induce the release of proinflammatory cytokines such as TNF-*α*, COX-2, iNOS, IL-6, and IL-1*β* [37–40]. The prolonged release of inflammatory cytokines in activated macrophages can result in uncontrolled inflammation, causing infection to the body [41]. Our study showed that the production of proinflammatory cytokines resulted in levels of TNF-*α*, iNOS, and IL-6 released from LPS-activated RAW264.7 cells being dose-dependently (100 and 200 *μ*g/ml) reduced in Tt-ME-treated groups (Figure 2(a)). Studies have shown that the NF-*κ*B and AP-1 transcription factors are coactivated by a variety of stimuli and activate gene transcription [42, 43]. Moreover, AP-1 subunits c-Fos and c-Jun physically interact with NF-*κ*B/p65 via the Rel homology domain [28]. The role of STAT3, NF-*κ*B, and AP-1 is to regulate key genes in inflammatory pathways such as angiogenesis and cell migration [44]. To confirm the effect of Tt-ME on transcription factor activity, luciferase assay was employed using plasmid constructs containing transcription factor-binding promoters. As a result, NF-*κ*B and AP-1 luciferase activity strongly decreased by Tt-ME in a dose-dependent manner (Figure 2(b)). Furthermore, we explored the effect of Tt-ME on nuclear translocation of NF-*κ*B subunits (p50 and p65), AP-1 subunits (c-Fos and c-Jun), and STAT3 in a time-dependent manner at 5, 15, 30, and 60 min. The inhibitory effect of Tt-ME on the translocation of nuclear proteins indicated that Tt-ME abrogates the effect of LPS on RAW264.7 cells by time-dependently inhibiting the nuclear translocation of p50, p65, c-Fos, c-Jun, and STAT3 (Figure 2(c)). This result implies that Tt-ME affects upstream signaling of the NF-*κ*B, AP-1, and STAT3 signaling pathways.

LPS induced NF-*κ*B activation, which phosphorylates the I*κ*B kinase complex and simultaneously degrades I*κ*B*α*, which results in translocation of p50/p65 NF-*κ*B subunits in the nucleus [45, 46]. Moreover, ERK1/2, JNK, and p38 have been identified as upstream subfamilies which activate AP-1 cascades [47, 48]. Therefore, to assess whether NF-*κ*B and AP-1 upstream signaling is involved in the anti-inflammatory effect of Tt-ME, we examined phosphorylation of I*κ*B*α*, p85, and AKT with respect to NF-*κ*B signaling (Figure 3(a)); ERK, JNK, and p38 with respect to AP-1 signaling; and JAK2 in a time-dependent manner (time ranging from 5 to 60 mins) (Figures 3(c) and 3(e)). Our results clearly demonstrate that Tt-ME strongly inhibited LPS-induced phosphorylation of I*κ*B*α*, p85, AKT, and ERK in a time-dependent manner at 5 min. However, Tt-ME did not inhibit JNK and p38 phosphorylation. These results suggest that Tt-ME selectively inhibited LPS-induced inflammatory signaling in RAW2647 macrophages. Our recent findings reported that Syk and Src contribute to NF-*κ*B-mediated phosphorylation of I*κ*B*α* at early time points [38, 49, 50]. Meanwhile, MEK1/2, MKK3/6, TAK1, and IRAK1 are known to be the upstream molecules of ERK, JNK, and p38 [51–53]. It seems clear that the activity of Syk and IRAK1 was found to be regulated by Tt-ME at 2 and 3 min, whereas Src, MEK1/2, MKK3/6, and TAK1 phosphorylation was inhibited by Tt-ME at 2, 3, and 5 mins in LPS-treated RAW264.7 cells (Figures 3(b) and 3(f)). These findings suggest that Tt-ME might target Src and Syk in NF-*κ*B and IRAK1 in AP-1 signaling because Syk and IRAK expression was found to be in greater proportion as reported previously [21, 54]. We corroborated the overexpression of Syk and IRAK1 in HEK293T cells and checked the downstream molecules of NF-*κ*B, AP-1, and STAT3 signaling in Tt-ME treatment. Syk overexpression highly regulated the phosphorylation of p65 and JAK2, whereas Tt-ME dose-dependently decreased phosphorylation with no effect on the total forms of Syk, p65, and JAK2 (Figure 3(d)). Similar results were observed in IRAK1 overexpression, which regulated the phosphorylation of c-Fos with no effect on total c-Fos levels (Figure 3(g)). Overall, our results confirm that Tt-ME targets Src, Syk, and IRAK1 to carry out its anti-inflammatory activity against NF-*κ*B, AP-1, and JAK2/STAT3 signaling.

Cell migration is one major phenomenon that plays an important role in inflammation. LPS enhanced TLR4 activation, which increased cell proliferation, invasion, and chemo resistance in HepG2 cells [55]. Studies have reported that LPS treatment enhances the migration of cells in RAW264.7 macrophages [56]. MMP-2 is the most important metalloproteinase, is involved in tumor migration, invasion, and metastasis, and is also known to regulate inflammation [57]. We first measured MMP-2 activity by Western blotting and found that it was time-dependently (0 to 60 min) increased in LPS-induced RAW264.7 cells. Similarly, LPS enhanced the migration of RAW264.7 cells, while Tt-ME-pretreated group (Figure 4(a)) inhibited migration of the cells (Figures 4(b) and 4(c)).

Gastric ulcers, the most common gastrointestinal disorder, are caused by an imbalance in acid, pepsin, *H. pylori*, and factors such as gastric mucus, bicarbonate ions, and prostaglandins, which leads to gastric lesions and cavities in the mucosa and tissue [58]. In Korea, medical expenditures related to gastritis and gastric ulcers range from $956.6 to $2553.10 per patient [59]. Moreover, traditional aromatic and herbal medicine has been used in Korea since ancient times to treat a variety of disorders including gastritis and ulcers [60]. We studied the effect of Tt-ME on HCl/EtOH-induced gastritis. In the present study, robust gastritis was observed with HCl/EtOH treatment, which leads to multiple band-like lesions in the gastric mucosa, whereas Tt-ME (200 mg/kg) and ranitidine (40 mg/kg) attenuated the gastric lesions (Figure 5(a)). Given the antigastritis role of Tt-ME, we used real-time PCR to examine cytokine gene expression and western blotting to examine inflammatory protein expression in relation to NF-*κ*B and STAT3 signaling pathways. Interestingly, EtOH/HCl-induced gastritis mouse models demonstrated an increase in IL-1*β*, iNOS, TNF-*α*, INF-*β*, and COX-2 gene expression and an increase in phosphorylation of p65, Syk, and JAK2. On the contrary, Tt-ME and ranitidine treatment decreased the gene expression of major cytokines, inflammatory mediators, and phosphorylation levels (Figures 5(b) and 5(c)). Taken together, these data suggest that EtOH/HCl increased gastric lesions, inflammatory gene expression, and protein expression, while the gastric lesions were significantly controlled by Tt-ME and ranitidine treatment.

**Figure 6:**
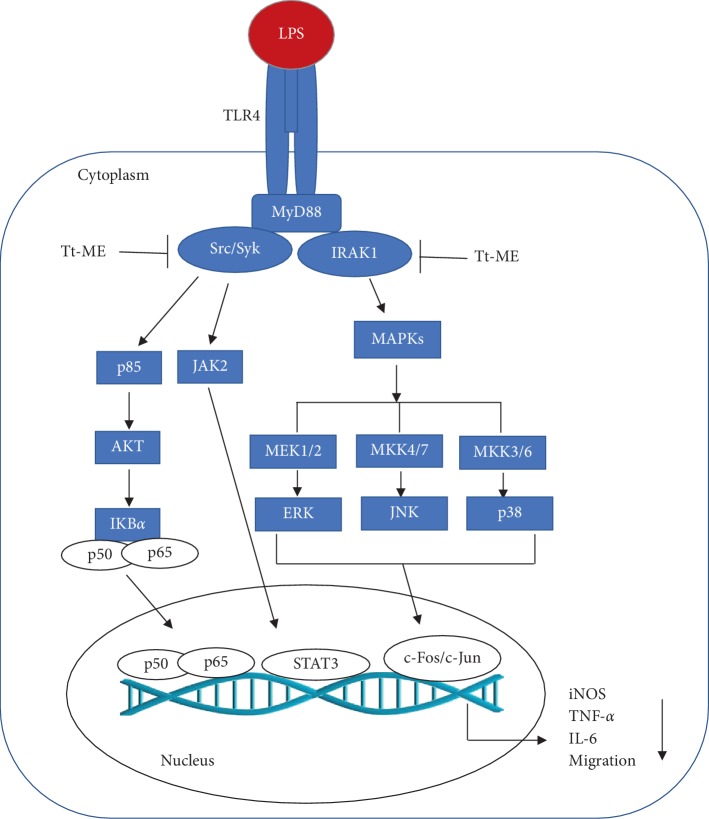
Inhibitory effect of Tt-ME in RAW264.7 macrophages. Schematic diagram representing the multi-inhibitory role of Tt-ME. Tt-ME treatment inhibits NF-*κ*B and the JAK2 signaling pathway by targeting Src, Syk tyrosine kinase, and IRAK1 kinase with respect to MAPK.

In conclusion, our study has provided evidence that Tt-ME is a potential antigastritis drug which targets Src, Syk, and IRAK1 and regulates putative anti-inflammatory pathways (Figure 6). Our study suggests Tt-ME as a preventive anti-inflammatory remedy. Nevertheless, more studies should be directed towards various other acute and chronic inflammatory diseases.

## Figures and Tables

**Table 1:** Semiquantitative PCR and real-time PCR primer sequences used in the study.

Primer name	Direction	Sequence (5′ to 3′)
*Semiquantitative PCR*		
iNOS	Forward	CCCTTCCGAAGTTTCTGGCAGCAG
	Reverse	GGCTGTCAGAGCCTCGTGGCTTTGG
IL-6	Forward	CACTACATCCTGACCCACTT
	Reverse	ATGCTCCTGCTTGAGTATGT
TNF-*α*	Forward	TTGACCTCAGCGCTGAGTTG
	Reverse	CCTGTAGCCCACGTCGTAGC
GAPDH	Forward	CACTCACGGCAAATTCAACGGCA
	Reverse	GACTCCACGACATACTCAGCAC

*Real-time PCR*		
iNOS	Forward	GGAGCCTTTAGACCTCAACAGA
	Reverse	TGAACGAGGAGGGTGGTG
COX-2	Forward	CACTACATCCTGACCCACTT
	Reverse	ATGCTCCTGCTTGAGTATGT
TNF-*α*	Forward	GCCTCTTCTCATTCCTGCTTG
	Reverse	CTGATGAGAGGGAGGCCATT
IFN-*β*	Forward	AAGAGTTACACTGCCTTTGCCATC
	Reverse	CACTGTCTGCTGGTGGAGTTCATC
IFN-*γ*	Forward	GGGTTGTTGACCTCAAACTTGGCA
	Reverse	CAGGCCATCAGCAACAACAT
IL1-*β*	Forward	CAACCAACAAGTGATATTCTCCATG
	Reverse	GATCCACACACTCCAGCTGCA
IL-6	Forward	CTAGGTTTGCCGAGTAGATCTC
	Reverse	GACAAAGCCAGAGTCCTTCAGAGA
iNOS	Forward	AACAATTCCTGGCGTTACCTT
	Reverse	CTGCCGTACAACTCCAGTGA
GAPDH	Forward	CAATGAATACGGCTACAGCAAC
	Reverse	AGGGAGATGCTCAGTGTTGG

## Data Availability

The data used to support the findings of this study are available from the corresponding author upon request.
