# Corrigendum to “*Trichosanthes tricuspidata* Lour. Methanol Extract Exhibits Anti-Inflammatory Activity by Targeting Syk, Src, and IRAK1 Kinase Activity”

**DOI:** 10.1155/2020/3183519

**Published:** 2020-12-02

**Authors:** Akash Ahuja, Deok Jeong, Mi-Yeon Kim, Jae Youl Cho

**Affiliations:** ^1^Department of Integrative Biotechnology, Sungkyunkwan University, Suwon 16419, Republic of Korea; ^2^School of Systems Biomedical Science, Soongsil University, Seoul 06978, Republic of Korea

In the article titled “*Trichosanthes tricuspidata* Lour. Methanol Extract Exhibits Anti-Inflammatory Activity by Targeting Syk, Src, and IRAK1 Kinase Activity” [1], there are concerns in relation to duplication in Figure 1(b), as raised on PubPeer [2]. The authors provided an explanation that they have mistakenly used the same migration images. The corrected figure and legend are presented here.

## Figures and Tables

**Figure 1:**
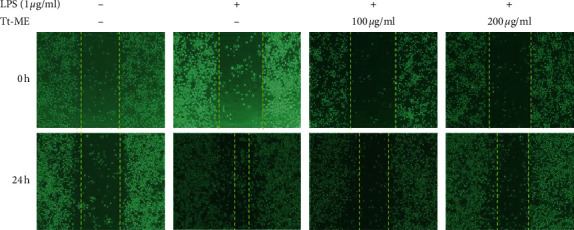
Tt-ME prevents LPS-induced migration abilities in RAW264.7 macrophages. (a) RAW264.7 macrophages were pretreated with Tt-ME (200 *μ*g/ml) for 30 minutes and then treated with LPS (1 *μ*g/ml) for the indicated period. Western blot analysis was performed to confirm MMP-2 levels. (b) RAW264.7 cells were scratched and then treated with Tt-ME for 30 minutes and then treated with LPS (1 *μ*g/ml) for 24 h. Representative images are shown at indicated time points. (c) Results were quantified using ImageJ software, and error bars represent SD of the means of three independent experiments performed in triplicates. Data are shown as the mean ± SD of three independent experiments. ^*∗*^*P* < 0.01; ^*∗∗*^*P* < 0.05.
